# Heredity, symptoms and risk factors of nasal polyps

**DOI:** 10.1186/2045-7022-5-S4-P24

**Published:** 2015-06-26

**Authors:** Anton Bohman, Martin Oscarsson, Mats Bende

**Affiliations:** 1 Uppsala University Hospital, Department of Ear, Nose and Throat Diseases, Uppsala, Sweden; 2 Hospital of Skaraborg, Skövde, Department of Otorhinolaryngology, Skövde, Sweden

## Background

The aim of this study was to investigate the heredity of nasal polyps and study the symptoms and risk factors of patients with this condition.

## Method

Patients with nasal polyps were recruited from the clinic, adult first-degree relatives of the patients were asked to participate. Our intention was to recruit one relative of each gender per patient. All participants were examined with nasal endoscopy and underwent a structured interview regarding upper airway symptoms and risk factors. The results were compared with a general population from a previous study who had been examined and questioned in the same way.

## Results

368 patients and 410 relatives were recruited. A control group, consisting of 1387 individuals from the general population, was used for comparison. The prevalence of nasal polyps among the relatives was 13.4%, which was almost five times higher than the prevalence in the control group (2.7%). The prevalence of nasal polyps within the families was 19.2%. The symptoms and risk factors associated with nasal polyps were nasal secretions, nasal blockage, sneezing and impaired sense of smell. Male sex, increasing age and asthma were also associated with the disease. Smoking was not a risk factor in this study.

## Conclusion

The results of this study strongly indicate that heredity is important in the development of nasal polyps. We are currently investigating possible genetic polymorphisms associated with nasal polyps in a genome-wide association study. Nasal secretions, nasal blockage, sneezing, impaired sense of smell, male sex, increasing age and asthma are symptoms and risk factors associated with nasal polyps.

